# Clinical analysis of preoperative deep vein thrombosis risk factors in patients with colorectal cancer: Retrospective observational study

**DOI:** 10.1002/ags3.12256

**Published:** 2019-04-29

**Authors:** Kazuya Nakagawa, Jun Watanabe, Yusuke Suwa, Shinsuke Suzuki, Atsushi Ishibe, Mitsuyoshi Ota, Chikara Kunisaki, Itaru Endo

**Affiliations:** ^1^ Department of Surgery Gastroenterological Center Yokohama City University Medical Center Yokohama Japan; ^2^ Department of Gastroenterological Surgery Yokohama City University Graduate School of Medicine Yokohama Japan

**Keywords:** colorectal cancer surgery, d‐dimer, deep vein thrombosis, risk factor, ultrasonography

## Abstract

**Aim:**

Deep vein thrombosis (DVT) is a major complication of cancer. The postoperative prevalence of DVT in colorectal cancer (CRC) surgery is high, but the preoperative prevalence and the risk factors have not been clarified in detail. The objective of this retrospective study was to investigate the preoperative prevalence and risk factors of DVT in patients admitted to hospital for CRC surgery.

**Methods:**

From January 2013 to March 2017, 1006 patients admitted for CRC surgery were deemed eligible for this retrospective study. Diagnosis of preoperative DVT was confirmed by compression ultrasonography. Prevalence of silent DVT in lower limbs in patients before CRC surgery was assessed, and the risk factors for preoperative DVT were investigated regarding the correlation of DVT with the patient's background.

**Results:**

Preoperative DVT and asymptomatic pulmonary thromboembolism were diagnosed in 136 (13.5%) and in 10 (1.0%) of 1006 patients overall, respectively. Multivariate analysis showed that increased age (≥75 years), female gender, and an elevated d‐dimer level (>1.0 μg/mL) were independent risk factors for preoperative DVT in this study. Notably, the prevalence of preoperative DVT exceeded 50% in patients with all three predictors.

**Conclusions:**

A high prevalence (13.5%) of preoperative DVT was found in patients admitted to the hospital for CRC surgery. The present results suggest that instrumental screening should be encouraged, at least in subgroups at a higher risk of preoperative DVT.

## 1 INTRODUCTION

Pulmonary thromboembolism (PE) is the second‐most common cause of death in patients with cancer,^1^, ^2^ and approximately 80% of PE result from deep venous thrombosis (DVT) of the lower extremities.^3^ Therefore, PE and DVT have been regarded as sequential conditions, and they are grouped together and generally called venous thromboembolism (VTE).

A prospective observational study in 2373 patients undergoing general, urological, or gynecological surgery reported that 50 patients (2.1%) were judged as affected by clinically overt VTE, and 12 events occurred within 5 days of surgery.^4^ Another report of the Japanese Society of Anesthesiologists in Japanese patients showed that postoperative VTE tended to occur on the first postoperative day in patients who had risk factors, such as malignant disease or obesity [5]. Fatal PE is known to primarily occur when getting out of bed for the first time after surgery. DVT causing such fatal PE may have already occurred before surgery, making postoperative anticoagulant therapy relatively ineffective in preventing such early fatal PE. Therefore, it is important to carry out preoperative screening for DVT.

There have been several reports of a high prevalence of DVT after abdominal cancer surgery in general.^6^, ^7^ Among patients with colorectal cancer, the estimated prevalence of DVT after surgery is 20%.^8^ However, the preoperative prevalence and the risk factors have not been clarified in detail. Only a few studies have focused on patients with gastroenterological cancer, although it is the most common risk factor for PE.^9^, ^10^, ^11^ To our knowledge, only one study with a small number of patients has been carried out in patients with colorectal cancer,^9^ and the covariates used to identify the independent risk factors were limited. In addition, the detailed anatomical distribution of DVT has not been reported.

Recently, lower‐extremity venous ultrasonography has been considered a useful method of diagnosing DVT because it is easily accessible, noninvasive, and has a high sensitivity (93%‐96%) and specificity (98%‐99%).^12^, ^13^, ^14^ The aim of the present study was to clarify the prevalence, anatomical distribution, and the risk factors for DVT of the lower extremities in patients with colorectal cancer before surgery.

## 2 MATERIALS AND METHODS

### 2.1 Patients

The study protocol was approved by the Ethical Advisory Committee of Yokohama City University School of Medicine. This retrospective study was registered with the Japanese Clinical Trials Registry as UMIN000033663 [http://www.umin.ac.jp/ctr/index.htm]. In this study, all processes complied with guidelines of the Declaration of Helsinki. From January 2013 to March 2017, a total of 1095 patients underwent CRC surgery at Yokohama City University Medical Center. Of these, 10 were excluded from the analysis because of emergency surgery, 54 were excluded because preoperative lower‐limb ultrasonography was not carried out, 21 were excluded because preoperative serum d‐dimer levels were not measured, and four were excluded because of postoperative hospital death (within 30 days). The remaining 1006 patients were enrolled in this retrospective study. Ultrasonography and serum d‐dimer level measurements were carried out as a preoperative examination within a mean of 4 weeks before CRC surgery.

Patients’ gender, weight, body mass index (BMI; weight in kilograms divided by the square of the height in meters), and data on the patients’ medical history and medical condition were recorded. TMN stages were recorded according to the 8th TMN classification of malignant tumors.^15^ Performance status (PS) was assessed using the scale of the Eastern Cooperative Oncology Group (ECOG).^16^

### 2.2 Diagnosis of preoperative DVT and PE

Compression ultrasonography (CUS) including the femoral, popliteal, and calf veins was carried out according to standard procedures (grayscale, B‐mode, color Doppler) preoperatively using a high‐end scanner (Logiq 7 Pro; GE Medical Systems, Milwaukee, WI, USA). In patients who underwent preoperative chemotherapy for rectal cancer, ultrasonography was done preoperatively. All examinations were carried out by one of several clinical technologists who were both trained in the performance of venous ultrasonography and certified as medical sonographers by the Japan Society of Ultrasonographics in Medicine. If a vein was distended by hypoechoic thrombus and showed partial or no compressibility without collaterals, we diagnosed acute DVT. If the vein was incompressible, narrow and irregular and showed echogenic thrombus attached to the venous walls with development of collaterals, we diagnosed chronic DVT.

To diagnose preoperative staging, contrast‐enhanced helical computed tomography (CT) was usually carried out if an iodine contrast agent was available. At the same time, we checked whether asymptomatic PE was present incidentally.

### 2.3 d‐Dimer assay

Blood samples for the d‐dimer analysis were obtained preoperatively. The samples were analyzed by the Nanopia d‐dimer assay (Sekisui Medical, Tokyo, Japan), which is the standard assay at Yokohama City University Medical Center. The Auto Dimer assay is a quantitative latex test for cross‐linked fibrin degradation products. All samples were handled according to the manufacturer's instructions. The samples were analyzed using an Automated Coagulation Analyzer CP3000 (Sekisui Medical). The technologists analyzing the samples were unaware of the CUS findings. Because the optimal cut‐off value of d‐dimer was unknown in preoperative DVT screening, the Yokohama City University Medical Center standard cut‐off level of 1.0 μg/mL was used.

### 2.4 Thromboprophylaxis

According to the Japanese Guidelines for Prevention of Venous Thromboembolism,^17^ most patients with colorectal cancer are classified in the high‐risk group for postoperative DVT. For high‐risk patients, physical treatments, such as intermittent pneumatic compression (IPC) or anticoagulant therapy, are recommended in the guidelines. Therefore, for patients in whom preoperative DVT was not detected, graduated compression stockings and IPC or anticoagulant therapy were carried out at the surgeon's discretion from the morning of surgery until the patient was able to walk adequately. Patients with distal DVT were generally given anticoagulant therapy using low‐molecular‐weight heparin (LMWH). For patients with proximal DVT, a temporary inferior vena cava filter (IVCF) was placed before surgery at the cardiologist's discretion.

### 2.5 Diagnosis of postoperative PE

When patients complained of symptoms such as dyspnea postoperatively, contrast‐enhanced helical CT was given to diagnose PE. Only those cases of PE that required some medical intervention (Common Terminology Criteria for Adverse Events grade 3 or higher^18^) were counted. Patients with asymptomatic postoperative PE were excluded from this study.

### 2.6 Statistical analyses

Continuous variables were presented as median (range) and compared using the Mann‐Whitney *U* test, whereas categorical variables were expressed as the absolute and relative frequencies and compared using the chi‐squared test.

Clinicopathological risk factors for preoperative DVT were primarily evaluated using univariate analyses. Variables that had relevant associations with preoperative DVT on these analyses (*P *<* *0.05) were included in a multivariate model. A multivariate analysis was carried out using logistic regression analysis. Backward elimination was used to select variable factors. Statistical significance was defined as *P* < 0.05. Analyses were done using the software package SPSS 22 (SPSS Inc., Chicago, IL, USA).

## 3 RESULTS

### 3.1 Characteristics of patients

A total of 1006 patients (595 men, 411 women) were included in this retrospective study. Median age of the patients was 69 years (range 27‐92 years). Clinicopathological characteristics of these patients are summarized in Table 1. There were 409 patients (40.7%) with rectal cancer. One hundred and forty‐five patients (14.4%) received preoperative chemotherapy. Of these, only seven patients used anti‐vascular endothelial growth factor (VEGF) agents. There were 101 patients (10.0%) who had simultaneous metastases.

**Table 1:** Characteristics of patients with colorectal cancer before surgery

Variable	*n*	%
Gender		
Male	595	59.1
Female	411	40.9
Age (years)		
<75	704	70.0
≧75	302	30.0
ECOG PS		
0	896	89.1
1/2/3	110	10.9
ASA		
1/2	927	92.1
3/4	79	7.9
Hypertension		
Present	426	42.3
Absent	580	57.7
Diabetes mellitus		
Present	182	18.1
Absent	824	81.9
Body mass index (kg/m^2^)		
<25	798	79.3
≧25	208	20.7
Location		
Colon	597	59.3
Rectum	409	40.7
cStage		
I/II/III	905	90.0
IV	101	10.0
Preoperative chemotherapy		
No	861	85.6
Yes	145	14.4
Central venous catheter		
Present	162	16.1
Absent	844	83.9
d‐Dimer (μg/mL)		
≦1.0	664	66.0
>1.0	342	34.0
CEA (ng/mL)		
<10	813	80.8
≧10	193	19.2
CA19‐9 (U/mL)		
<100	962	95.6
≧100	44	4.4

ASA, American Society of Anesthesiologists; CA19‐9, carbohydrate antigen 19‐9; CEA, carcinoembryonic antigen; ECOG, Eastern Cooperative Oncology Group; PS, performance status.

### 3.2 Prevalence and anatomical distribution of DVT

Of the 1006 patients, 136 (13.5%) were found to have DVT preoperatively, and all patients with DVT were asymptomatic. Fifteen patients had proximal DVT (thrombosis involving the popliteal vein and above), and 121 patients had distal DVT only. The anatomical distribution of DVT is shown in Table 2. The most common site of DVT was the soleal veins. In only 41 of 136 patients were the DVT diagnosed as chronic by preoperative ultrasonography.

**Table 2:** Anatomical distribution of deep venous thrombosis in patients with colorectal cancer before surgery

Distribution of DVT	Right	Left	Total	
Distal type			121	12.0%
Soleal vein	60	67		
Posterior tibial vein	5	7		
Peroneal vein	6	8		
Gastrocnemius vein	0	0		
Small saphenous vein	0	3		
Proximal type			15	1.5%
Popliteal vein	3	5		
Superficial femoral vein	3	3		
Deep femoral vein	0	0		
Common femoral vein	1	0		
External iliac vein	0	0		
Internal iliac vein	0	0		

DVT, deep venous thrombosis.

### 3.3 Prevalence of preoperative PE and incidence of postoperative VTE

By contrast‐enhanced helical CT to diagnose preoperative staging, asymptomatic PE was detected in 10 patients (1.0%). We detected simultaneous DVT in eight patients by ultrasonography. Four patients had proximal DVT, and four had distal DVT. Preoperative anticoagulant therapy was carried out in nine patients, and retrievable IVCF was combined for one patient. None of the 10 patients with postoperative symptomatic VTE developed fatal PE.

Of the 15 patients with proximal DVT, preoperative anticoagulant therapy was carried out in 12. Of these, two patients had a retrievable IVCF placed. No preoperative therapy was carried out in three patients because of chronic DVT only or dementia. Symptomatic postoperative PE did not occur in any case, and thrombosis in IVCF occurred in one patient. By continuing anticoagulant therapy, the IVCF was able to be removed.

Of the 121 patients with distal DVT, preoperative anticoagulant therapy was carried out in 40. Of these 40 patients, unfractionated heparin or LMWH was used for 27, and direct oral anticoagulant was used for 13. Preoperative anticoagulant therapy was not done in 81 due to the existence of chronic DVT only, risk of bleeding from the tumor, and doctor's judgment that treatment was not necessary.

Symptomatic postoperative PE occurred in one patient: a 79‐year‐old woman with distal DVT. Her preoperative d‐dimer level was 2.0 μg/mL. She received no preoperative anticoagulant therapy because of the risk of bleeding from the tumor. On the first postoperative day, she was able to get out of bed with no complaints of dyspnea. However, she needed oxygen because of reduced saturation. On the second postoperative day, she complained of dyspnea when washing her face in the morning. CT showed shadow defects at the secondary bifurcation of the pulmonary artery. We diagnosed her with postoperative PE. A retrievable IVCF was placed, and anticoagulant therapy was started, after which her symptoms improved. On the 20th postoperative day, she was discharged while on anticoagulant therapy.

### 3.4 Risk factors for preoperative DVT

Clinicopathological factors and the presence or absence of DVT are summarized in Table 3. Of the 136 patients who were found to have DVT, 56 were male, and 80 were female. DVT were found in 24.2% of elderly patients (≥75 years of age) and in 21.8% of patients with PS ≥1. Similarly, 15.8% of patients with cStageIV and 17.2% of patients with a history of preoperative chemotherapy were found to have DVT. Univariate analysis showed that the incidence of DVT was significantly higher in women, patients ≥75 years of age, those with PS ≥1, colon cancer patients, and in those with elevated d‐dimer levels (>1.0 μg/mL).

**Table 3:** Incidence of deep venous thrombosis according to patient background

Variable	DVT (‐)		DVT (+)		*P* value
	*n*	%	*n*	%	
Gender					
Male	539	90.6	56	9.4	<0.01
Female	331	80.5	80	19.5	
Age (years)					
<75	641	91.1	63	8.9	<0.01
≧75	229	75.8	73	24.2	
ECOG PS					
0	784	87.5	112	12.5	0.01
1/2/3	86	78.2	24	21.8	
ASA					
1/2	805	86.8	122	13.2	0.30
3/4	65	82.3	14	17.7	
Hypertension					
Present	358	84.0	68	16.0	0.06
Absent	512	88.3	68	11.7	
Diabetes mellitus					
Present	158	86.8	24	13.2	1.00
Absent	712	86.4	112	13.6	
Body mass index (kg/m^2^)					
<25	692	86.7	106	13.3	0.65
≧25	178	85.6	30	14.4	
Location					
Colon	504	84.4	93	15.6	0.02
Rectum	366	89.5	43	10.5	
cStage					
I/II/III	785	86.7	120	13.3	0.45
IV	85	74.2	16	15.8	
Preoperative chemotherapy					
No	750	87.1	111	12.9	0.19
Yes	120	82.8	25	17.2	
Central venous catheter					
Present	133	82.1	29	17.9	0.08
Absent	737	87.3	107	12.7	
d‐Dimer (μg/mL)					
≦1.0	628	94.6	36	5.4	<0.01
>1.0	242	70.8	100	29.2	
CEA (ng/mL)					
<10	705	86.7	108	13.3	0.64
≧10	165	75.5	28	14.5	
CA19‐9 (U/mL)					
<100	834	86.7	128	13.3	0.37
≧100	36	81.8	8	18.2	

ASA, American Society of Anesthesiologists; CA19‐9, carbohydrate antigen 19‐9; CEA, carcinoembryonic antigen; DVT, deep venous thrombosis; ECOG, Eastern Cooperative Oncology Group; PS, performance status.

Results of univariate and multivariate analyses of risk factors for preoperative DVT are shown in Table 4. The factors with a *P* value <0.05 in the univariate analysis were subjected to a multivariate analysis by entering them into a logistic regression model using backward elimination to determine the independent predictors of the risk of preoperative DVT. The multivariate analysis showed that female gender (*P *<* *0.01; odds ratio [OR] 2.5; 95% confidence interval [CI] 1.4‐3.8), increased age (≥75 years) (*P < *0.01; OR 2.1; 95% CI 1.4‐3.1), and elevated d‐dimer levels (>1.0 μg/mL) (*P *<* *0.01; OR 6.3; 95% CI 4.1‐9.6) were independent risk factors for preoperative DVT.

**Table 4:** Univariate and multivariate analyses of predictive factors for preoperative DVT

Variable	Univariate			Multivariate		
	OR	95% CI	*P* value	OR	95% CI	*P* value
Gender, female	2.3	1.6‐3.4	<0.01	2.5	1.4‐3.8	<0.01
Age, ≧75 years	3.2	2.2‐4.70	<0.01	2.1	1.4‐3.1	<0.01
PS(ECOG), ≧1	2.0	1.2‐3.2	<0.01			
Location, colon	1.6	1.1‐2.3	<0.01			
d‐Dimer, >1.0 μg/mL	7.2	4.8‐10.9	<0.01	6.3	4.1‐9.6	<0.01

CI, confidence interval; DVT, deep venous thrombosis; ECOG, Eastern Cooperative Oncology Group; OR, odds ratio; PS, performance status.

Of the 136 patients who were found to have DVT, 15 had proximal‐type DVT, six of whom had chronic DVT only. Univariate analysis showed that the incidence of proximal DVT was significantly higher in patients with elevated d‐dimer levels (>1.0 μg/mL) and in the presence of a central venous catheter. On multivariate analysis, only elevated d‐dimer levels (>1.0 μg/mL) (*P *<* *0.01; OR 5.5; 95% CI 1.7‐17.4) were found to be an independent risk factor for preoperative proximal DVT.

### 3.5 Risk stratification of preoperative DVT using independent predictors

**Figure 1:**
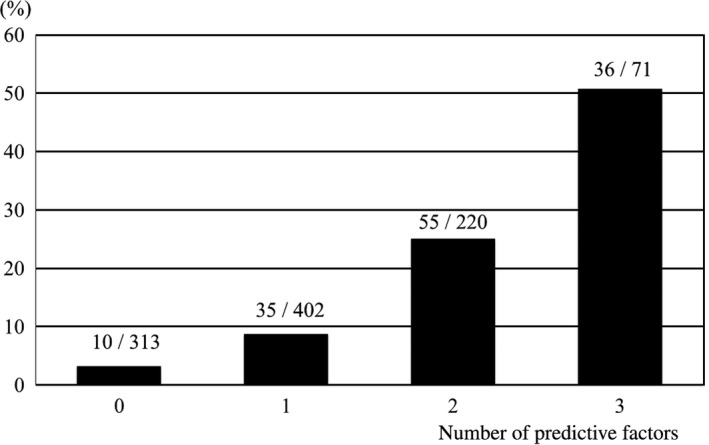
Incidence of preoperative deep venous thrombosis increased according to the number of independent predictors

When the patients were divided into four groups according to the accompanying number of independent predictors for preoperative DVT, the prevalence rate was 3.2% (10/313), 8.7% (35/402), 25.0% (55/220), and 50.7% (36/71) in patients with 0, 1, 2, and 3 predictors, respectively. This indicates that the more accompanying independent predictors, the higher the incidence of preoperative DVT (Figure 1). Notably, the prevalence of preoperative DVT exceeded 50% in women ≥75 years of age with elevated d‐dimer levels (>1.0 μg/mL).

## 4 DISCUSSION

We analyzed a total of 1006 patients who underwent elective colorectal resection. Preoperative DVT was diagnosed in 136 patients (13.5%). Multivariate analysis showed that increased age (≥75 years), female gender, and elevated d‐dimer levels (>1.0 μg/mL) were independent risk factors for preoperative DVT.

A few studies have reported the prevalence and risk factors of preoperative DVT in patients with gastroenterological cancer. However, only the study of Stender et al specifically reported these values in colorectal cancer patients.^9^ They reported the prevalence and risk factors of DVT in 193 patients with colorectal cancer. They showed that DVT was detected in 15 patients (7.8%), and the risk of DVT was strongly correlated with female gender and elevated American Society of Anesthesiologists (ASA) risk score. Although female gender was also a risk factor for DVT on multivariate analysis in the present study, the ASA risk score was not a risk factor. Of note, however, the study of Stender et al was a relatively small‐scale study and the covariates used to identify the independent risk factors were limited.

Recently, Tanizawa et al.^10^ reported that of 1140 patients with gastric cancer, 86 (7.5%) had DVT preoperatively. In their study, female gender, older age, worse PS, presence of a central venous catheter, and a history of preoperative chemotherapy were independent risk factors for DVT. Although the patients’ diagnoses differed between their study and ours (gastric and colorectal cancer, respectively), we similarly found that female gender and older age were independent risk factors for DVT. Wada et al.^11^ reported that of 976 patients with gastric cancer, preoperative DVT was diagnosed by lower‐extremity ultrasonography in 13 (1.3%), and neoadjuvant chemotherapy was a risk factor for preoperative detection of DVT. They carried out lower‐extremity ultrasonography in patients with a positive d‐dimer assay result. In the present study, the prevalence of DVT was higher than that in the study of Wada et al, possibly as a result of differences in the study design and in the characteristics of patients.

We showed that older age (≥75 years) was an independent risk factor for preoperative DVT. Several authors have reported that the prevalence of VTE is greater in older patients than in younger ones.^10^, ^19^, ^20^ In Japan, Wakabayashi et al. analyzed 505 patients who underwent total hip arthroplasty and investigated the risk factors for preoperative VTE. One of the factors significantly related to preoperative VTE was increased age.^21^ Furthermore, Tanizawa et al.^10^ analyzed 1140 patients who underwent gastric cancer surgery and showed that age ≥80 years was an independent risk factor for preoperative DVT. Consistent with previous reports, in the present study, the DVT‐positive patients were significantly older than the DVT‐negative patients.

In the present study, we also showed that elevated d‐dimer levels (>1.0 μg/mL) were an independent risk factor for preoperative DVT. d‐Dimer is a degradation product of crosslinked fibrin that appears in the blood after a blood clot is degraded by fibrinolysis.^22^ Elevated d‐dimer levels in the blood predict increased secondary fibrinolytic activity and are a principal marker of hypercoagulation and thrombosis.^23^, ^24^, ^25^ d‐Dimer levels are elevated in the setting of acute deep vein thrombosis,^26^ and normal levels are expected in the absence of acute deep vein thrombosis unless other coexistent conditions that activate the coagulation system are present.^27^, ^28^ The d‐dimer assay is a safe and useful tool with a high sensitivity (97%‐100%) for excluding acute DVT and a high negative predictive value (96%‐100%).^29^, ^30^ Therefore, our finding that elevated d‐dimer levels (>1.0 μg/mL) were an independent risk factor for preoperative DVT was an expected result.

Lower‐extremity venous ultrasonography has a high sensitivity (93%‐96%) as well as high specificity (98%‐99%) for the diagnosis of DVT.^12^, ^13^, ^14^ However, ultrasonography in all preoperative patients as a screening tool is considered to represent overuse of this tool.^31^, ^32^ The appropriate use of the d‐dimer assay can limit the overuse and added cost of ultrasonography without any negative impact. 31 Wada et al. reported that their preoperative DVT screening method using the d‐dimer assay in combination with ultrasonography seemed to be effective for detecting DVT in gastric cancer patients scheduled for surgery. However, we must emphasize that the d‐dimer level may not be elevated in cases of chronic thrombus, as d‐dimer is a degradation product of crosslinked fibrin. In our series of 136 patients with DVT, 41 (30.1%) had chronic DVT, and 20 (48.8%) of these patients had normal d‐dimer levels.

Although a medical history of VTE is a reported risk factor for postoperative VTE, the clinical significance of asymptomatic chronic thrombosis is unknown. Therefore, whether or not we need to detect asymptomatic chronic thrombosis in preoperative screening tests is unknown. Of the six patients who had proximal chronic DVT in the present study, four had a normal level of serum d‐dimer. These patients seem to have a higher risk of postoperative VTE than those without DVT. Tanizawa et al.^10^ reported that DVT increased or a new DVT was detected in five of 17 patients who had chronic DVT before surgery. Although the appropriate use of the d‐dimer assay can limit the overuse and added cost of ultrasonography, further studies of asymptomatic chronic DVT are needed.

The significance of preoperative VTE is still debatable. In the present study, postoperative PE occurred in only one patient with preoperative DVT. There was no difference in postoperative VTE occurrence between patients with and without preoperative DVT. However, a medical history of VTE is a reported risk factor for postoperative VTE. The presence of preoperative DVT is also considered a risk factor and, to prevent postoperative VTE, it is important to screen preoperative DVT patients and to take appropriate measures such as anticoagulant therapy. In this retrospective study, we had no uniform treatment strategy for preoperative anticoagulant therapy in patients with VTE. If preoperative VTE was detected, we consulted a cardiologist on a case‐by‐case basis and the cardiologist decided the treatment plan. In this study, not all patients with preoperative VTE received anticoagulant therapy. Although it increases the risk of bleeding from the tumor, we think preoperative anticoagulant therapy is preferred to prevent postoperative PE. If a patient has chronic DVT only, it is unclear whether anticoagulant therapy is needed.

Several limitations associated with the present study warrant mention. First, this was a retrospective study conducted at a single institution. Second, the incidence of postoperative asymptomatic DVT was unclear, as postoperative lower‐extremity ultrasonography was not routinely done, being carried out only in patients who developed postoperative symptomatic PE. Third, although the results of this study may be generalizable to Japanese patients, their generalizability to patients in other countries, especially Western countries, is uncertain.

In conclusion, a high prevalence (13.5%) of preoperative DVT was found in patients admitted to hospital for colorectal cancer surgery. The present results suggest that instrumental screening should be encouraged at least in subgroups at an increased risk of preoperative DVT.

## DISCLOSURE

Conflicts of Interest: Authors declare no conflicts of interest for this article.

## AUTHOR CONTRIBUTION

Funding: Authors received no funding for this study.

Kazuya Nakagawa designed the study, and wrote the initial draft of manuscript. Jun Watanabe, Chikara Kunisaki, and Itaru Endo contributed to analysis and interpretation of data, and assisted in the preparation of the manuscript. Yusuke Suwa, Shinsuke Suzuki, Atsushi Ishibe, and Mitsuyoshi Ota have contributed to data collection and interpretation, and critically reviewed the manuscript. All authors approved the final version of the manuscript.
